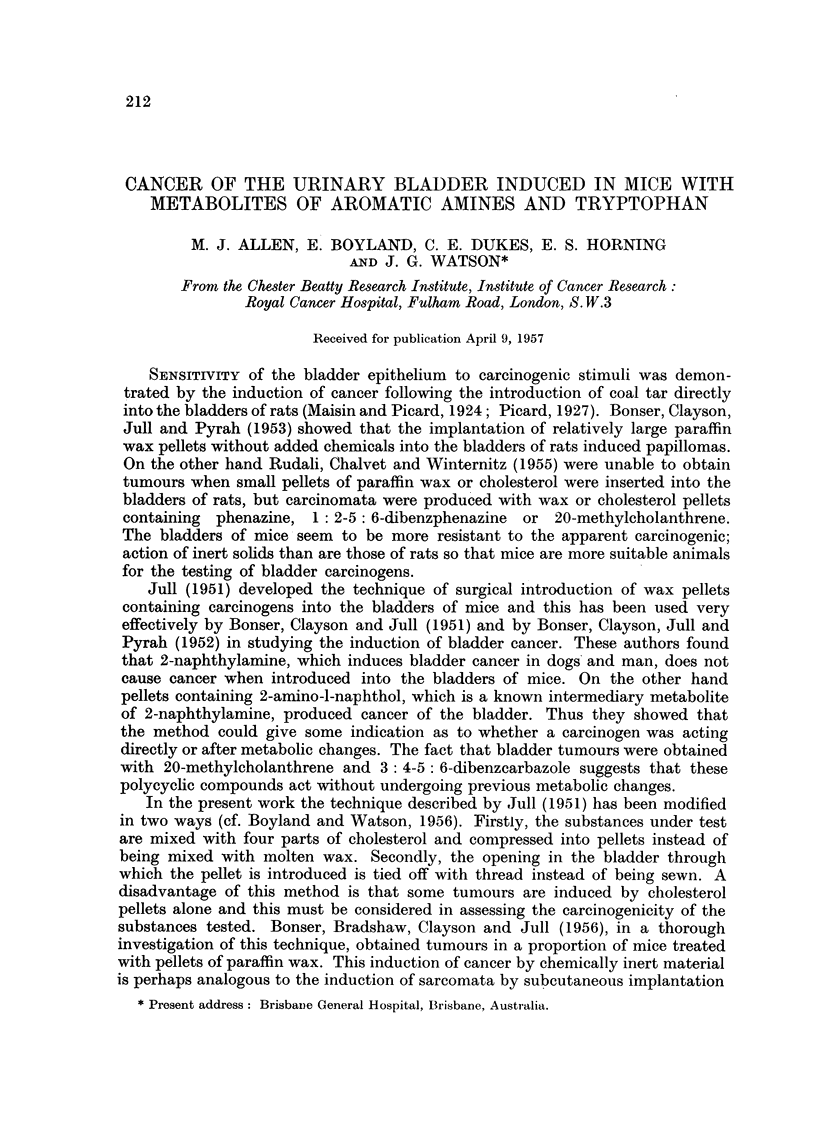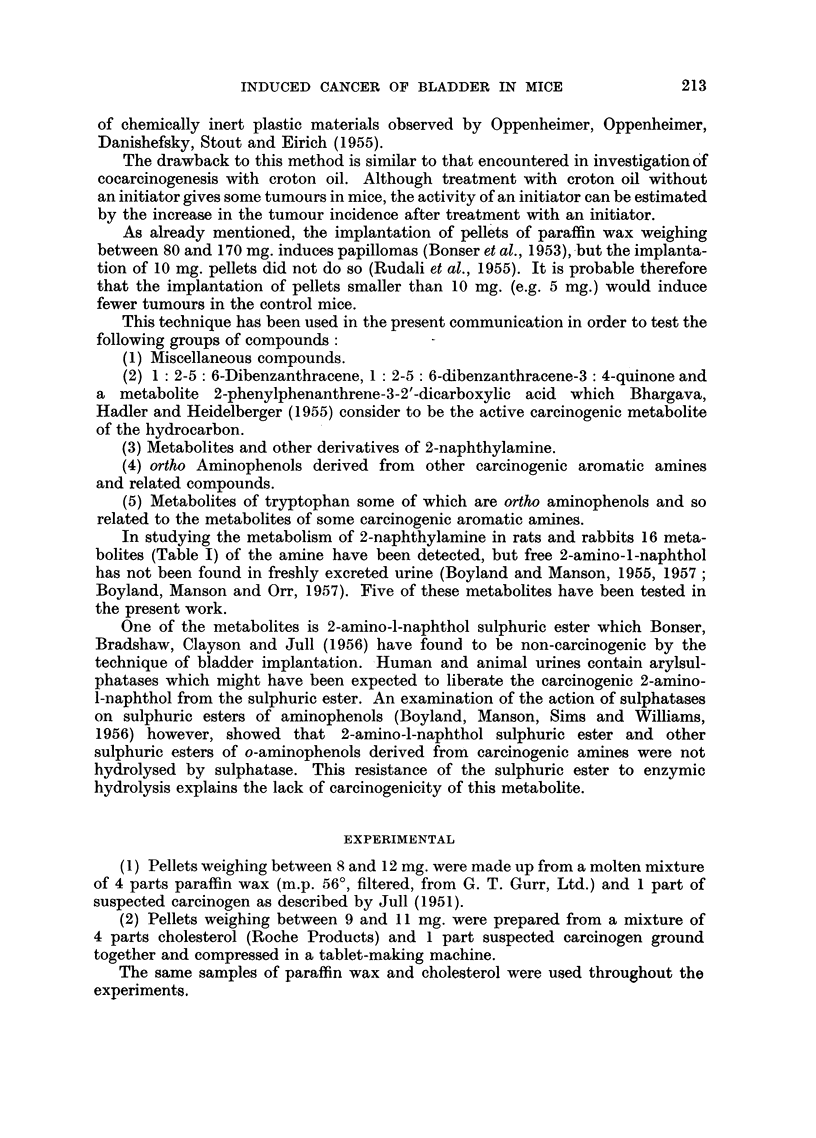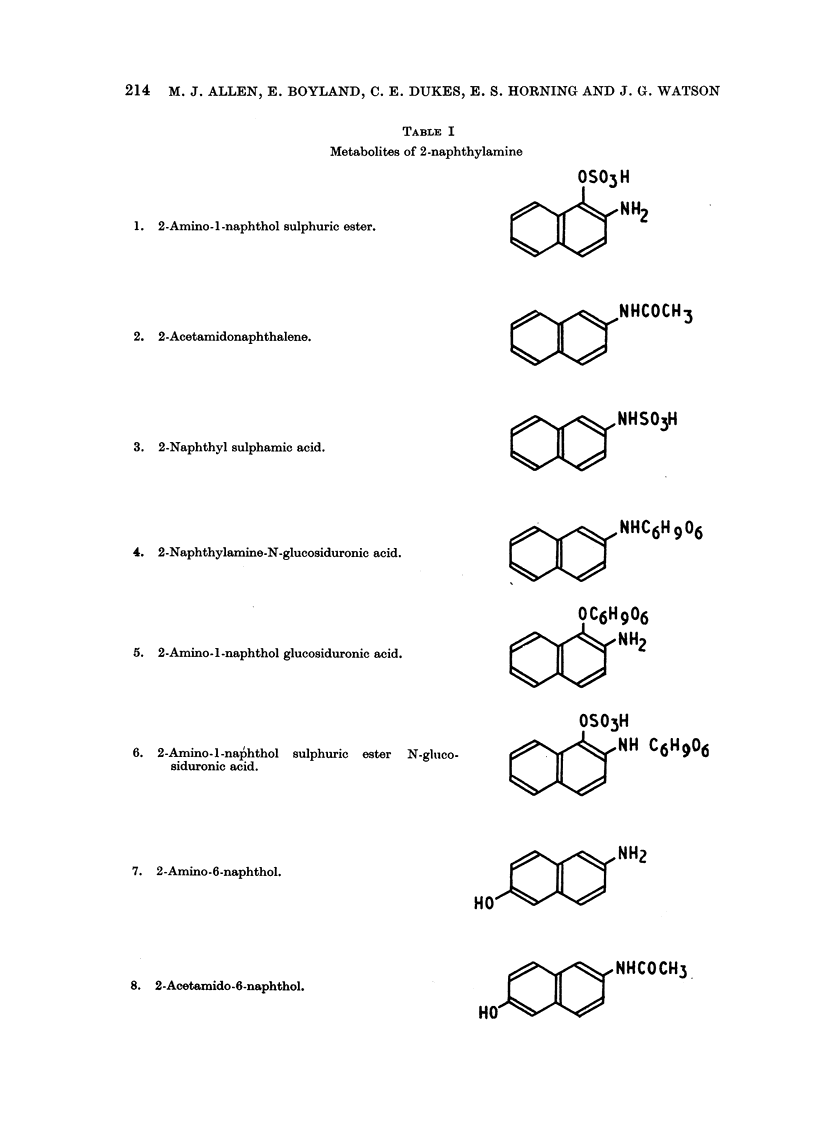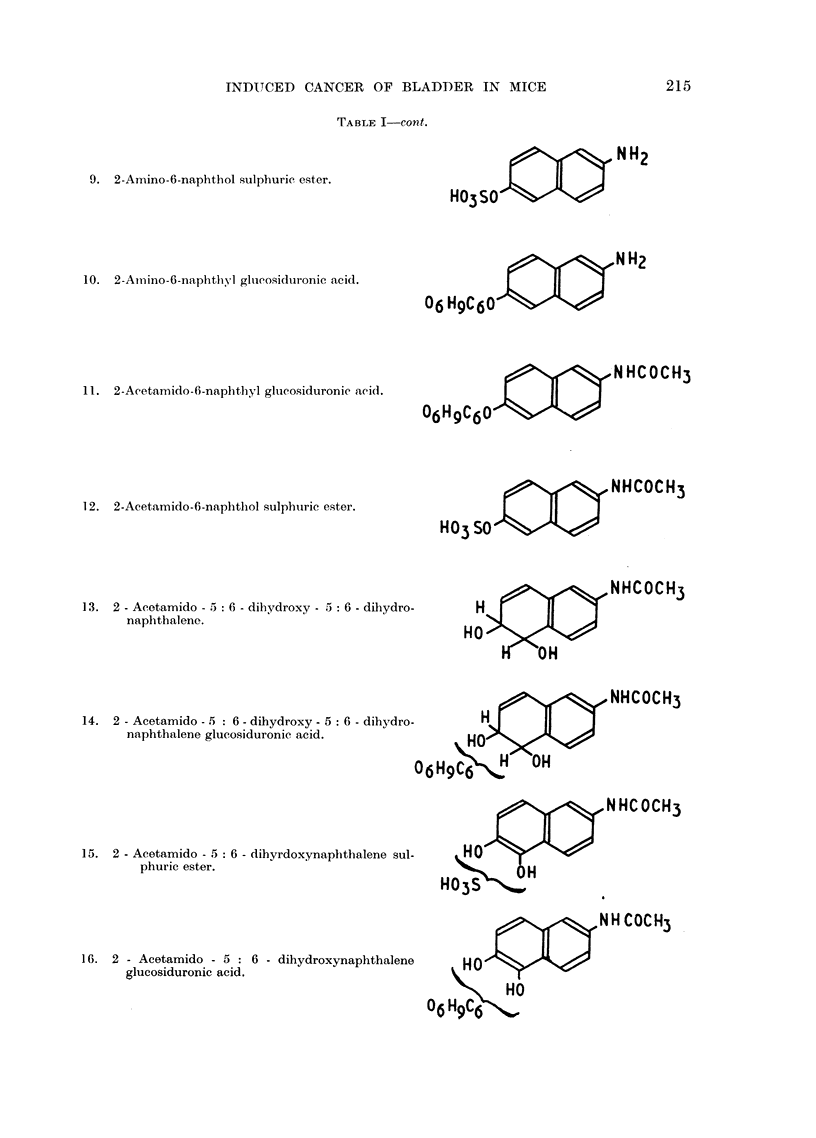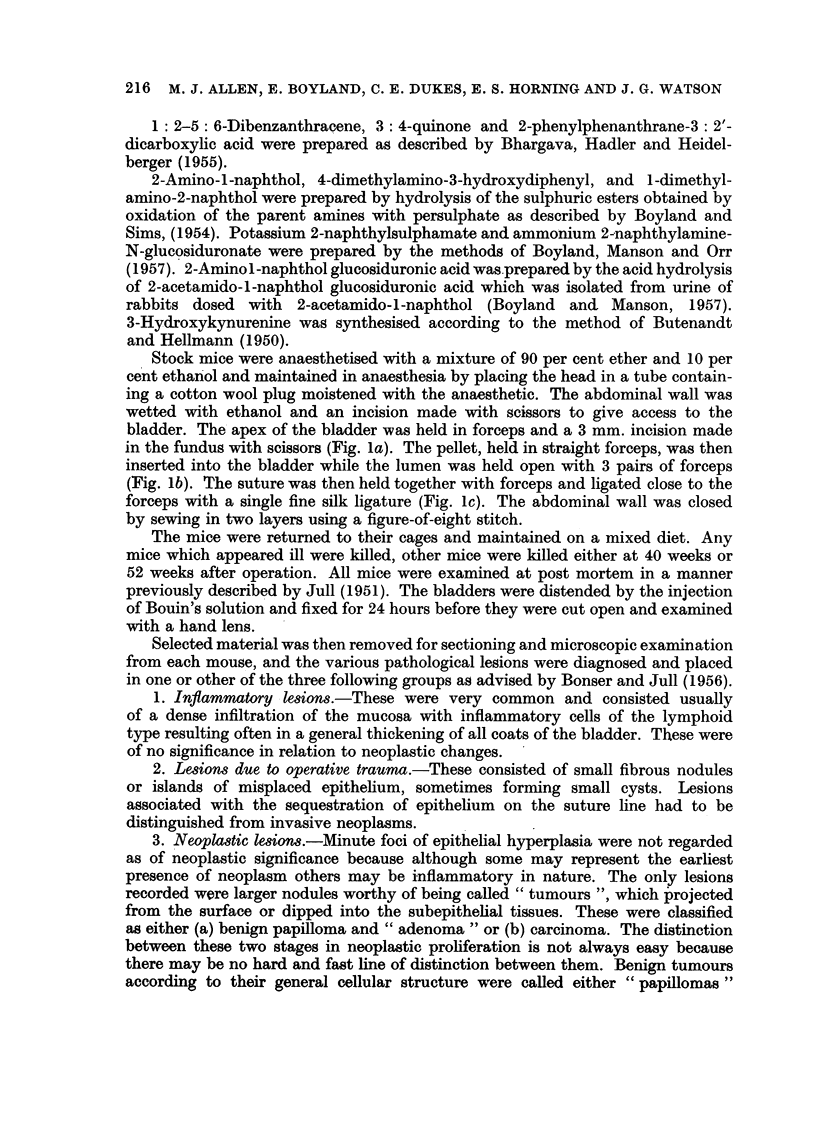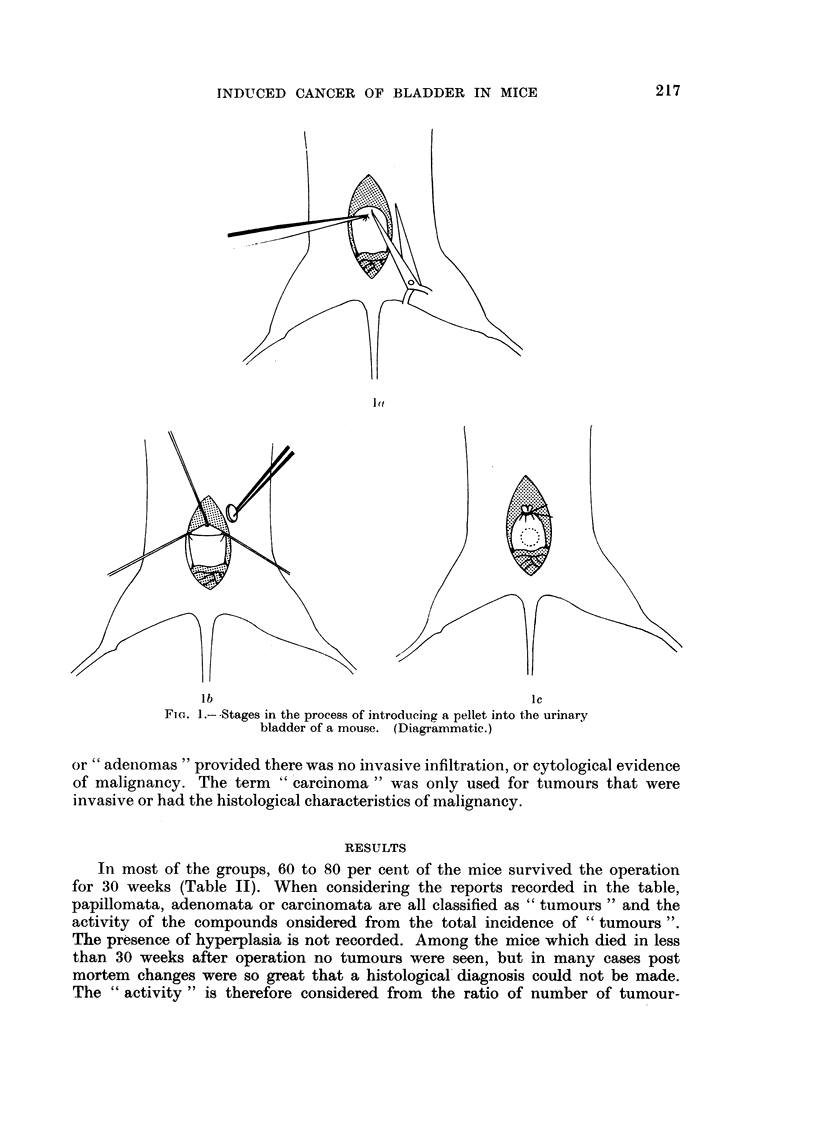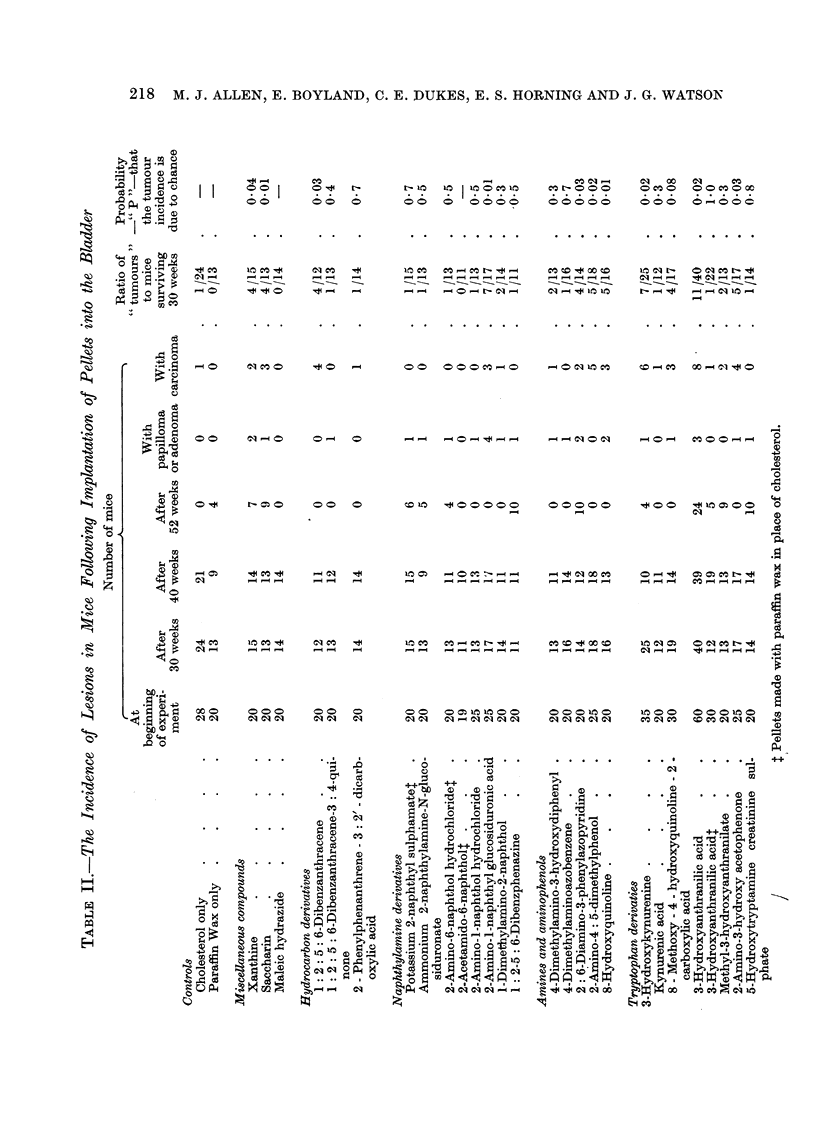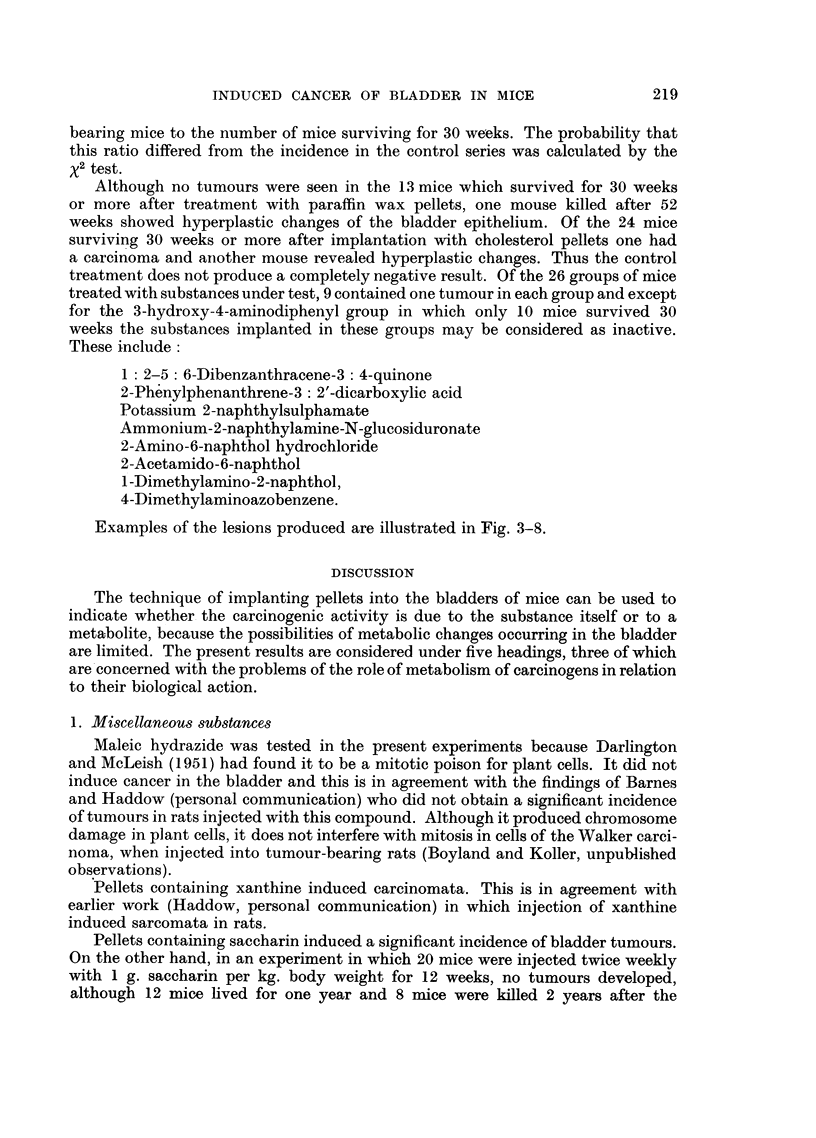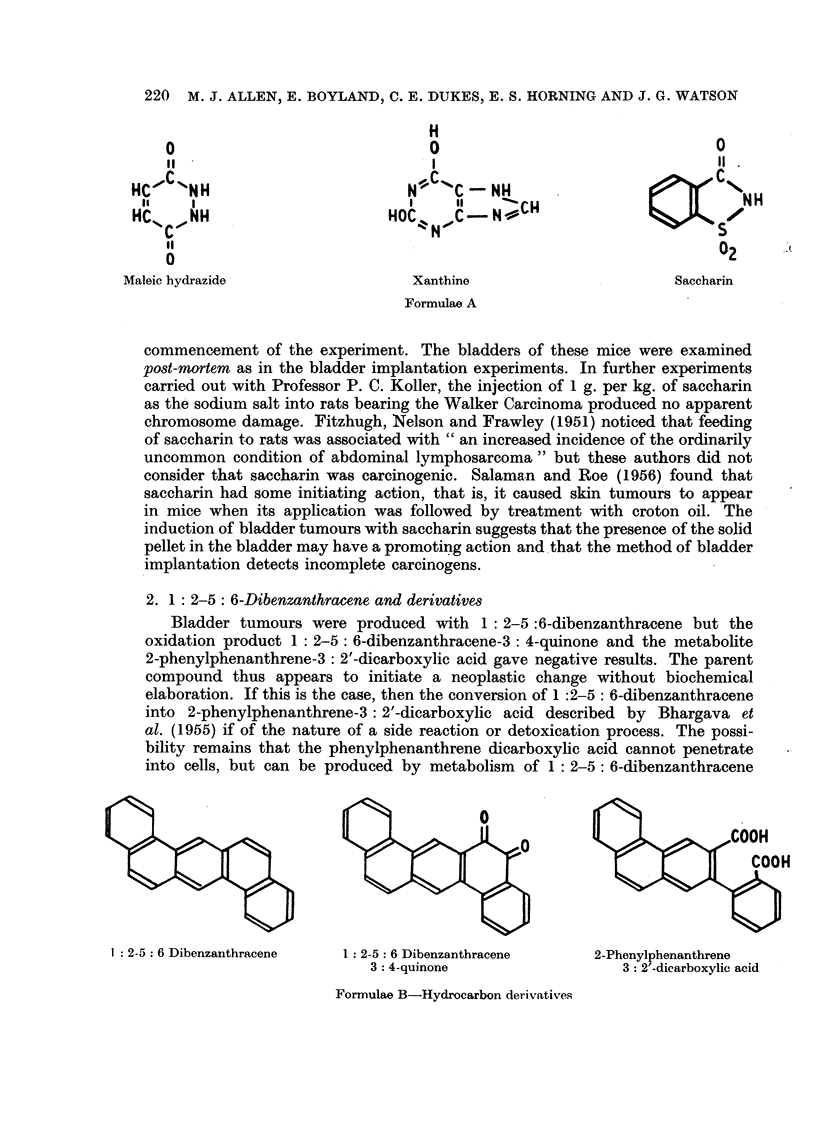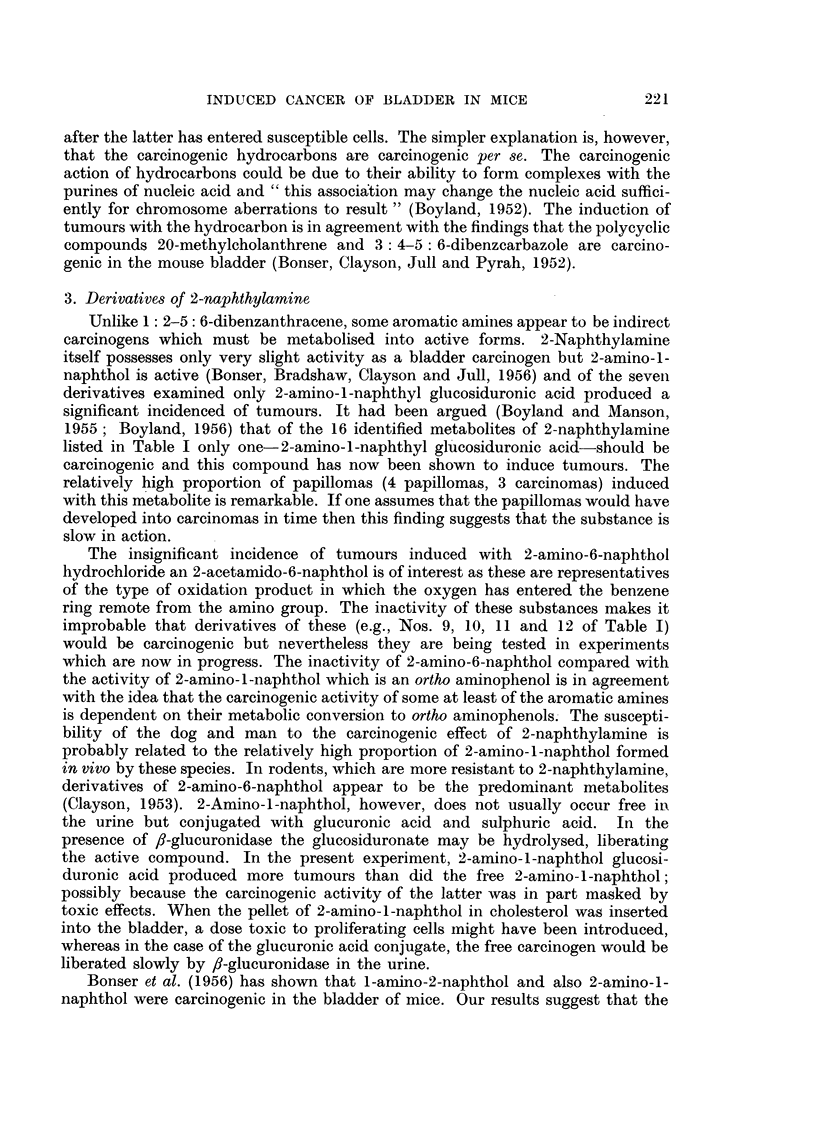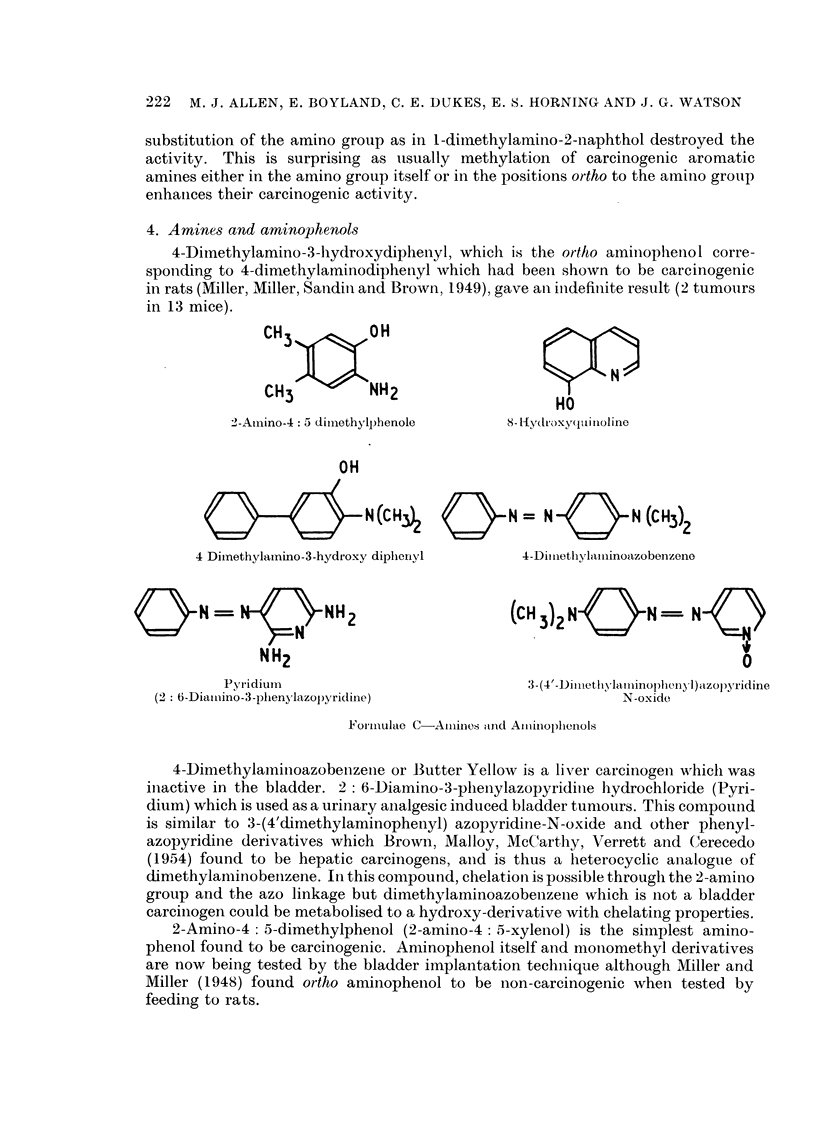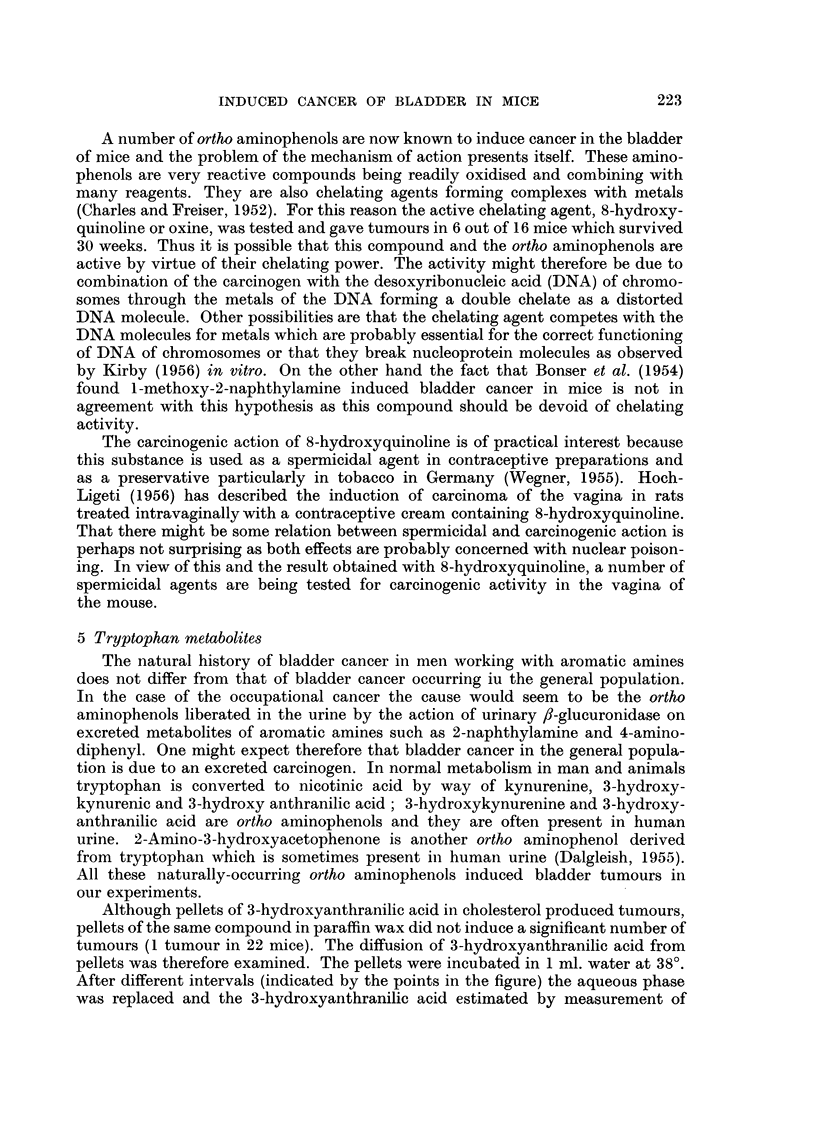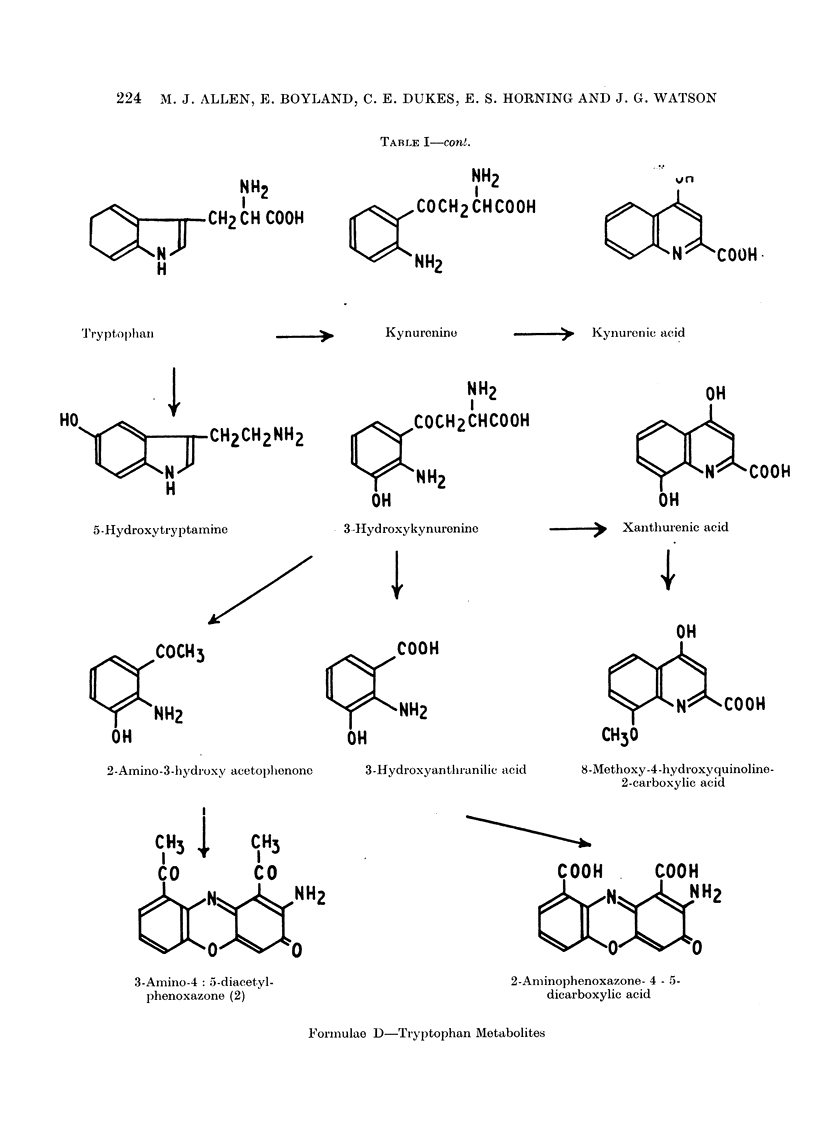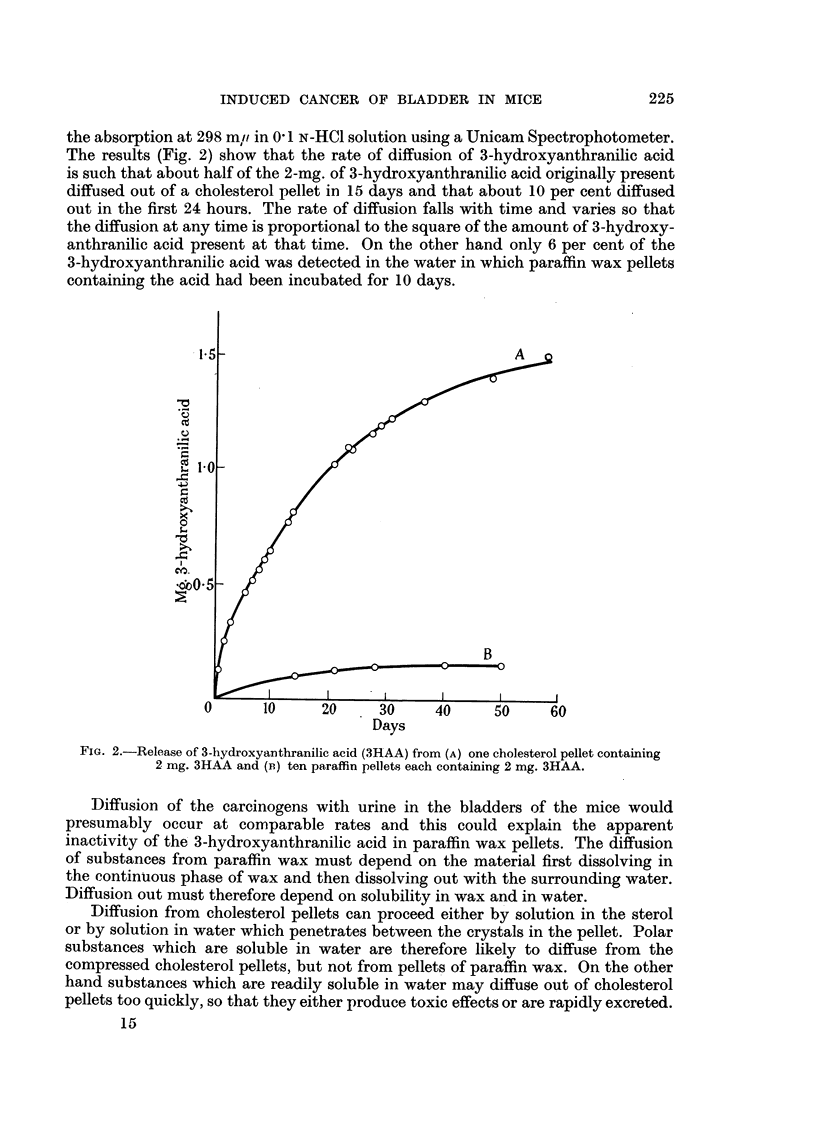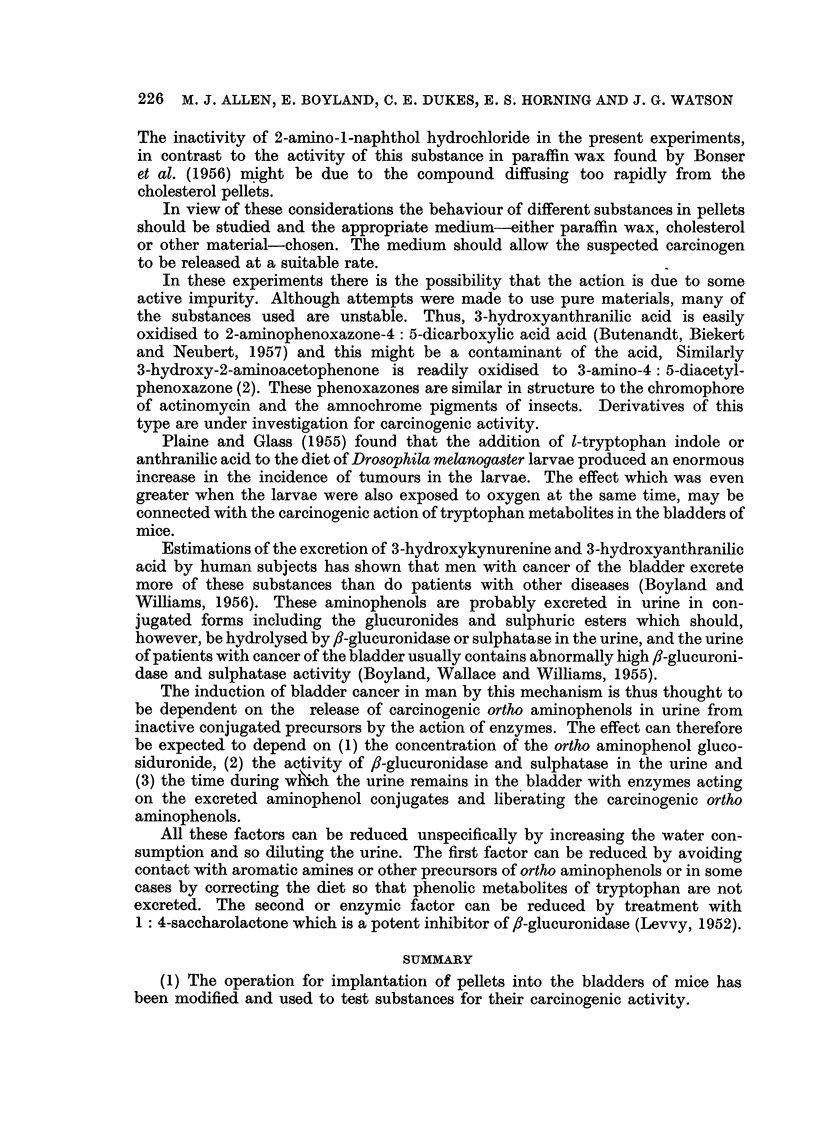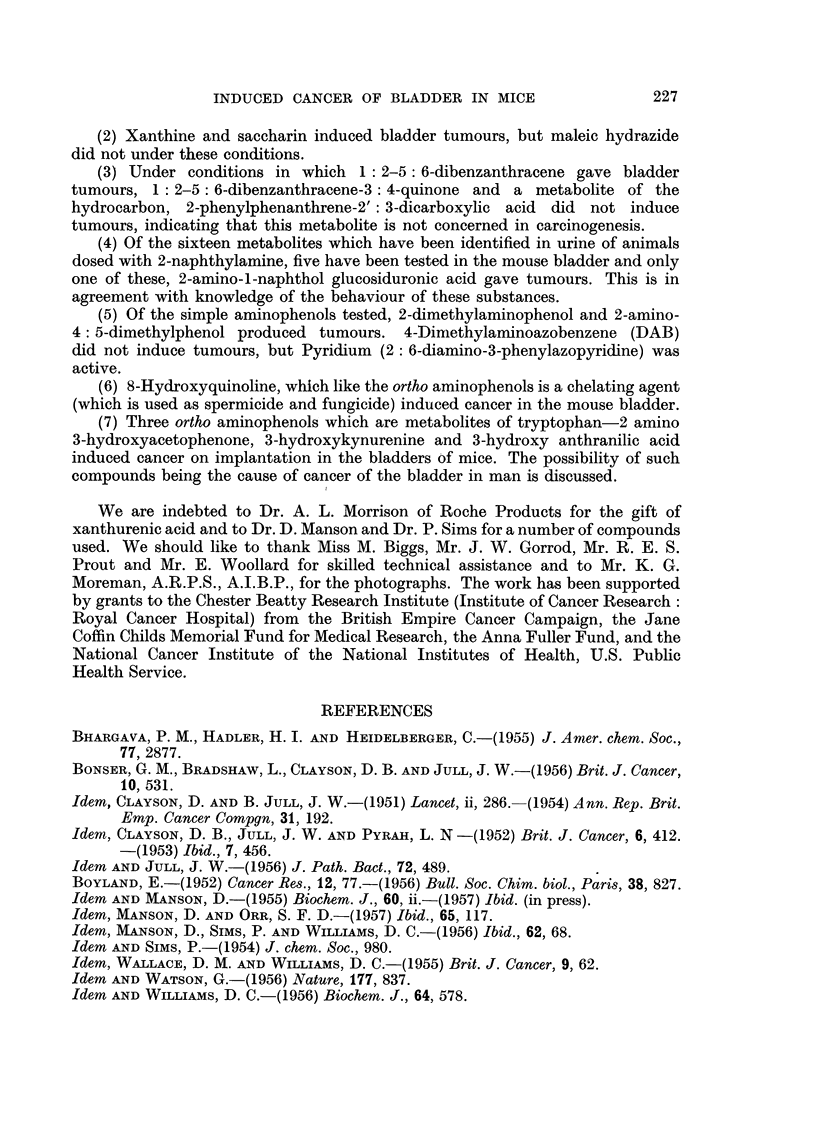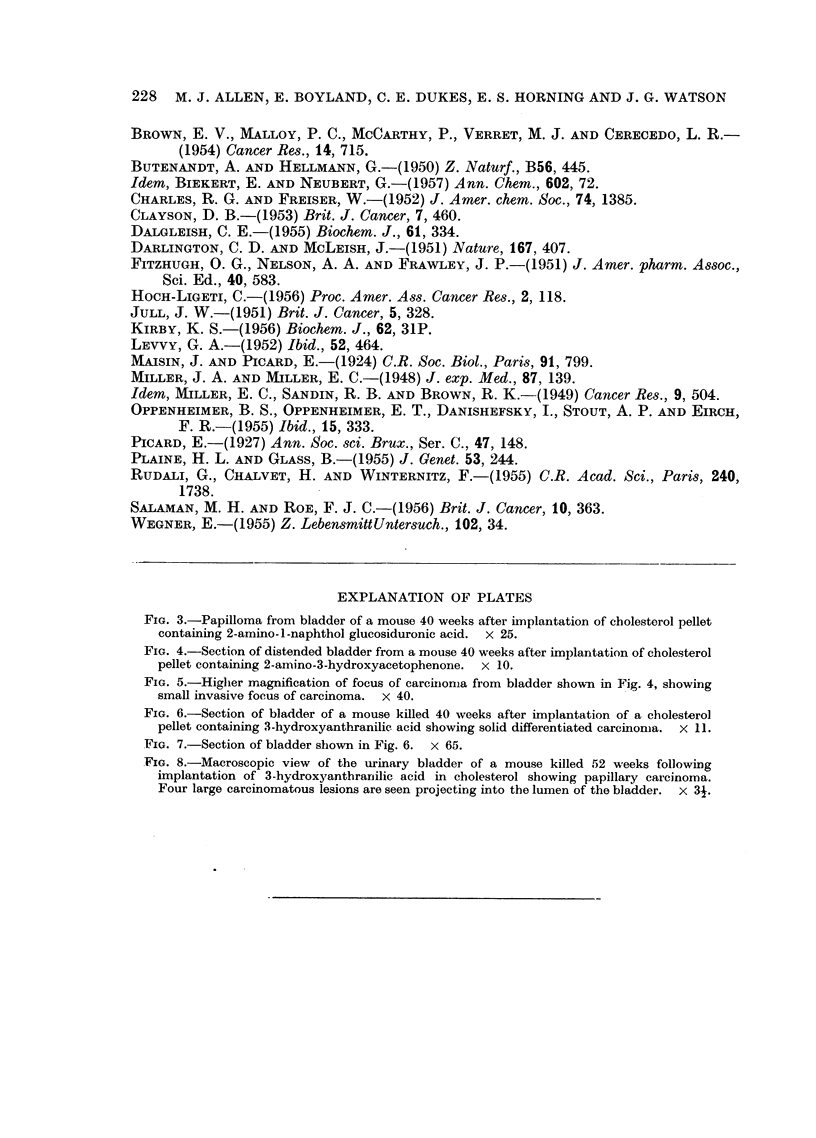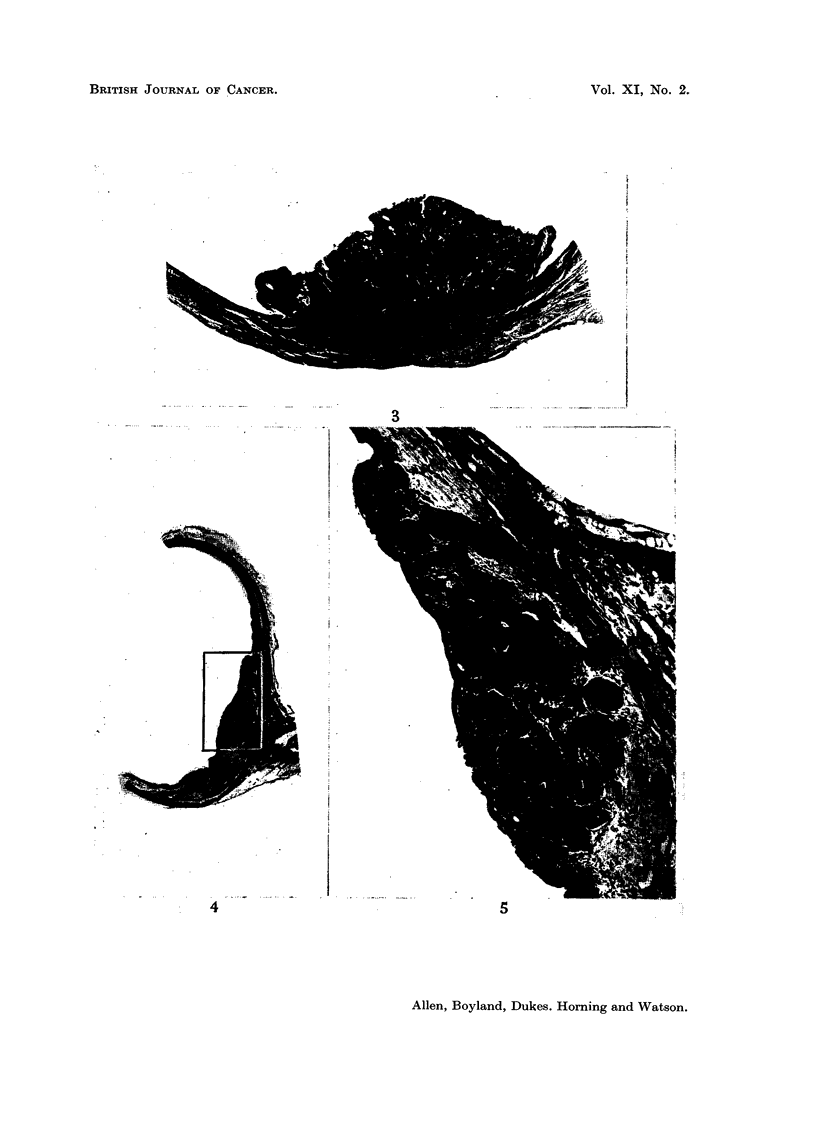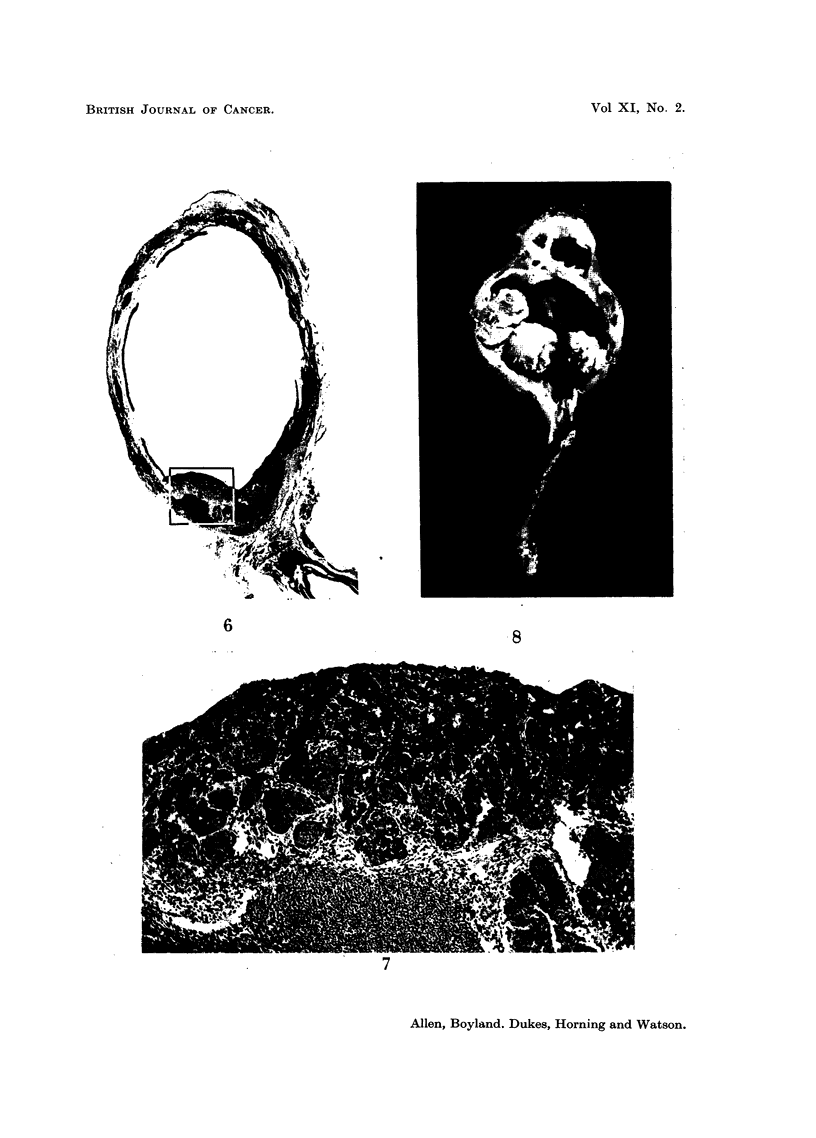# Cancer of the Urinary Bladder Induced in Mice with Metabolites of Aromatic Amines and Tryptophan

**DOI:** 10.1038/bjc.1957.29

**Published:** 1957-06

**Authors:** M. J. Allen, E. Boyland, C. E. Dukes, E. S. Horning, J. G. Watson

## Abstract

**Images:**


### CANCER OF THE URINARY BLADDER INDUCED IN MICE WITH METABOLITES OF AROMATIC AMINES AND TRYPTOPHAN

M. J. ALLEN, E. BOYLAND, C. E. DUKES, E. S. HORNING AND J. G. WATSON*

*From the Chester Beatty Research Institute, Institute of Cancer Research : Royal Cancer Hospital, Fulham Road, London, S.W.3*

Received for publication April 9, 1957

SENSITIVITY of the bladder epithelium to carcinogenic stimuli was demontrated by the induction of cancer following the introduction of coal tar directly into the bladders of rats (Maisin and Picard, 1924 ; Picard, 1927). Bonser, Clayson, Jull and Pyrah (1953) showed that the implantation of relatively large paraffin wax pellets without added chemicals into the bladders of rats induced papillomas. On the other hand Rudali, Chalvet and Winternitz (1955) were unable to obtain tumours when small pellets of paraffin wax or cholesterol were inserted into the bladders of rats, but carcinomata were produced with wax or cholesterol pellets containing phenazine, 1 : 2-5 : 6-dibenzphenazine or 20-methylcholanthrene. The bladders of mice seem to be more resistant to the apparent carcinogenic; action of inert solids than are those of rats so that mice are more suitable animals for the testing of bladder carcinogens.

Jull (1951) developed the technique of surgical introduction of wax pellets containing carcinogens into the bladders of mice and this has been used very effectively by Bonser, Clayson and Jull (1951) and by Bonser, Clayson, Jull and Pyrah (1952) in studying the induction of bladder cancer. These authors found that 2-naphthylamine, which induces bladder cancer in dogs and man, does not cause cancer when introduced into the bladders of mice. On the other hand pellets containing 2-amino-l-naphthol, which is a known intermediary metabolite of 2-naphthylamine, produced cancer of the bladder. Thus they showed that the method could give some indication as to whether a carcinogen was acting directly or after metabolic changes. The fact that bladder tumours were obtained with 20-methylcholanthrene and 3 : 4-5 : 6-dibenzcarbazole suggests that these polycyclic compounds act without undergoing previous metabolic changes.

In the present work the technique described by Jull (1951) has been modified in two ways (cf. Boyland and Watson, 1956). Firstly, the substances under test are mixed with four parts of cholesterol and compressed into pellets instead of being mixed with molten wax. Secondly, the opening in the bladder through which the pellet is introduced is tied off with thread instead of being sewn. A disadvantage of this method is that some tumours are induced by cholesterol pellets alone and this must be considered in assessing the carcinogenicity of the substances tested. Bonser, Bradshaw, Clayson and Jull (1956), in a thorough investigation of this technique, obtained tumours in a proportion of mice treated with pellets of paraffin wax. This induction of cancer by chemically inert material is perhaps analogous to the induction of sarcomata by subcutaneous implantation

* Present address : Brisbane General Hospital, Brisbane, Australia.

of chemically inert plastic materials observed by Oppenheimer, Oppenheimer, Danishefsky, Stout and Eirich (1955).

The drawback to this method is similar to that encountered in investigation of cocarcinogenesis with croton oil. Although treatment with croton oil without an initiator gives some tumours in mice, the activity of an initiator can be estimated by the increase in the tumour incidence after treatment with an initiator.

As already mentioned, the implantation of pellets of paraffin wax weighing between 80 and 170 mg. induces papillomas (Bonser *et al.*, 1953), but the implantation of 10 mg. pellets did not do so (Rudali *et al.*, 1955). It is probable therefore that the implantation of pellets smaller than 10 mg. (e.g. 5 mg.) would induce fewer tumours in the control mice.

This technique has been used in the present communication in order to test the following groups of compounds :

(1) Miscellaneous compounds.

(2) 1 : 2-5 : 6-Dibenzanthracene, 1 : 2-5 : 6-dibenzanthracene-3 : 4-quinone and a metabolite 2-phenylphenanthrene-3-2′-dicarboxylic acid which Bhargava, Hadler and Heidelberger (1955) consider to be the active carcinogenic metabolite of the hydrocarbon.

(3) Metabolites and other derivatives of 2-naphthylamine.

(4) *ortho* Aminophenols derived from other carcinogenic aromatic amines and related compounds.

(5) Metabolites of tryptophan some of which are *ortho* aminophenols and so related to the metabolites of some carcinogenic aromatic amines.

In studying the metabolism of 2-naphthylamine in rats and rabbits 16 metabolites (Table I) of the amine have been detected, but free 2-amino-1-naphthol has not been found in freshly excreted urine (Boyland and Manson, 1955, 1957 ; Boyland, Manson and Orr, 1957). Five of these metabolites have been tested in the present work.

One of the metabolites is 2-amino-l-naphthol sulphuric ester which Bonser, Bradshaw, Clayson and Jull (1956) have found to be non-carcinogenic by the technique of bladder implantation. Human and animal urines contain arylsulphatases which might have been expected to liberate the carcinogenic 2-amino-l-naphthol from the sulphuric ester. An examination of the action of sulphatases on sulphuric esters of aminophenols (Boyland, Manson, Sims and Williams, 1956) however, showed that 2-amino-l-naphthol sulphuric ester and other sulphuric esters of *o*-aminophenols derived from carcinogenic amines were not hydrolysed by sulphatase. This resistance of the sulphuric ester to enzymic hydrolysis explains the lack of carcinogenicity of this metabolite.

### EXPERIMENTAL

(1) Pellets weighing between 8 and 12 mg. were made up from a molten mixture of 4 parts paraffin wax (m.p. 56°, filtered, from G. T. Gurr, Ltd.) and 1 part of suspected carcinogen as described by Jull (1951).

(2) Pellets weighing between 9 and 11 mg. were prepared from a mixture of 4 parts cholesterol (Roche Products) and 1 part suspected carcinogen ground together and compressed in a tablet-making machine.

The same samples of paraffin wax and cholesterol were used throughout the experiments.

TABLE I

Metabolites of 2-naphthylamine

1. 2-Amino-1-naphthol sulphuric ester.

2. 2-Acetamidonaphthalene.

3. 2-Naphthyl sulphamic acid.

4. 2-Naphthylamine-N-glucosiduronic acid.

5. 2-Amino-1-naphthol glucosiduronic acid.

6. 2-Amino-1-naphthol sulphuric ester N-glucosiduronic acid.

7. 2-Amino-6-naphthol.

8. 2-Acetamido-6-naphthol.

TABLE I—*cont.*

9. 2-Amino-6-naphthol sulphuric ester.

10. 2-Amino-6-naphthyl glucosiduronic acid.

11. 2-Acetamido-6-naphthyl glucosiduronic acid.

12. 2-Acetamido-6-naphthol sulphuric ester.

13. 2 - Acetamido - 5 : 6 - dihydroxy - 5 : 6 - dihydro-naphthalene.

14. 2 - Acetamido - 5 : 6 - dihydroxy - 5 : 6 - dihydro-naphthalene glucosiduronic acid.

15. 2 - Acetamido - 5 : 6 - dihyrdoxynaphthalene sul-phuric ester.

16. 2 - Acetamido - 5 : 6 - dihydroxynaphthalene glucosiduronic acid.

1 : 2–5 : 6-Dibenzanthracene, 3 : 4-quinone and 2-phenylphenanthrane-3 : 2′-dicarboxylic acid were prepared as described by Bhargava, Hadler and Heidelberger (1955).

2-Amino-1-naphthol, 4-dimethylamino-3-hydroxydiphenyl, and 1-dimethylamino-2-naphthol were prepared by hydrolysis of the sulphuric esters obtained by oxidation of the parent amines with persulphate as described by Boyland and Sims, (1954). Potassium 2-naphthylsulphamate and ammonium 2-naphthylamine-N-glucosiduronate were prepared by the methods of Boyland, Manson and Orr (1957). 2-Amino1-naphthol glucosiduronic acid was prepared by the acid hydrolysis of 2-acetamido-1-naphthol glucosiduronic acid which was isolated from urine of rabbits dosed with 2-acetamido-1-naphthol (Boyland and Manson, 1957). 3-Hydroxykynurenine was synthesised according to the method of Butenandt and Hellmann (1950).

Stock mice were anaesthetised with a mixture of 90 per cent ether and 10 per cent ethanol and maintained in anaesthesia by placing the head in a tube containing a cotton wool plug moistened with the anaesthetic. The abdominal wall was wetted with ethanol and an incision made with scissors to give access to the bladder. The apex of the bladder was held in forceps and a 3 mm. incision made in the fundus with scissors (Fig. 1*a*). The pellet, held in straight forceps, was then inserted into the bladder while the lumen was held open with 3 pairs of forceps (Fig. 1*b*). The suture was then held together with forceps and ligated close to the forceps with a single fine silk ligature (Fig. 1*c*). The abdominal wall was closed by sewing in two layers using a figure-of-eight stitch.

The mice were returned to their cages and maintained on a mixed diet. Any mice which appeared ill were killed, other mice were killed either at 40 weeks or 52 weeks after operation. All mice were examined at post mortem in a manner previously described by Jull (1951). The bladders were distended by the injection of Bouin's solution and fixed for 24 hours before they were cut open and examined with a hand lens.

Selected material was then removed for sectioning and microscopic examination from each mouse, and the various pathological lesions were diagnosed and placed in one or other of the three following groups as advised by Bonser and Jull (1956).

1. *Inflammatory lesions.*—These were very common and consisted usually of a dense infiltration of the mucosa with inflammatory cells of the lymphoid type resulting often in a general thickening of all coats of the bladder. These were of no significance in relation to neoplastic changes.

2. *Lesions due to operative trauma.*—These consisted of small fibrous nodules or islands of misplaced epithelium, sometimes forming small cysts. Lesions associated with the sequestration of epithelium on the suture line had to be distinguished from invasive neoplasms.

3. *Neoplastic lesions.*—Minute foci of epithelial hyperplasia were not regarded as of neoplastic significance because although some may represent the earliest presence of neoplasm others may be inflammatory in nature. The only lesions recorded were larger nodules worthy of being called " tumours ", which projected from the surface or dipped into the subepithelial tissues. These were classified as either (a) benign papilloma and " adenoma " or (b) carcinoma. The distinction between these two stages in neoplastic proliferation is not always easy because there may be no hard and fast line of distinction between them. Benign tumours according to their general cellular structure were called either " papillomas "

1a

1b 1c

FIG. 1.—Stages in the process of introducing a pellet into the urinary bladder of a mouse. (Diagrammatic.)

or " adenomas " provided there was no invasive infiltration, or cytological evidence of malignancy. The term " carcinoma " was only used for tumours that were invasive or had the histological characteristics of malignancy.

### RESULTS

In most of the groups, 60 to 80 per cent of the mice survived the operation for 30 weeks (Table II). When considering the reports recorded in the table, papillomata, adenomata or carcinomata are all classified as " tumours " and the activity of the compounds onsidered from the total incidence of " tumours ". The presence of hyperplasia is not recorded. Among the mice which died in less than 30 weeks after operation no tumours were seen, but in many cases post mortem changes were so great that a histological diagnosis could not be made. The " activity " is therefore considered from the ratio of number of tumour-

TABLE II.—*The Incidence of Lesions in Mice Following Implantation of Pellets into the Bladder*

| | Number of mice | | | | | | | |
|---|---|---|---|---|---|---|---|---|
| | At beginning of experiment | After 30 weeks | After 40 weeks | After 52 weeks | With papilloma or adenoma | With carcinoma | Ratio of "tumours" to mice surviving 30 weeks | Probability —"P"—that the tumour incidence is due to chance |
| *Controls* | | | | | | | | |
| Cholesterol only | 28 | 24 | 21 | 0 | 0 | 1 | 1/24 | — |
| Paraffin Wax only | 20 | 13 | 9 | 4 | 0 | 0 | 0/13 | — |
| *Miscellaneous compounds* | | | | | | | | |
| Xanthine | 20 | 15 | 14 | 7 | 2 | 2 | 4/15 | 0·04 |
| Saccharin | 20 | 13 | 13 | 9 | 1 | 3 | 4/13 | 0·01 |
| Maleic hydrazide | 20 | 14 | 14 | 0 | 0 | 0 | 0/14 | — |
| *Hydrocarbon derivatives* | | | | | | | | |
| 1 : 2 : 5 : 6-Dibenzanthracene | 20 | 12 | 11 | 0 | 0 | 4 | 4/12 | 0·03 |
| 1 : 2 : 5 : 6-Dibenzanthracene-3 : 4-quinone | 20 | 13 | 12 | 0 | 1 | 0 | 1/13 | 0·4 |
| 2 - Phenylphenanthrene - 3 : 2′ - dicarboxylic acid | 20 | 14 | 14 | 0 | 0 | 1 | 1/14 | 0·7 |
| *Naphthylamine derivatives* | | | | | | | | |
| Potassium 2-naphthyl sulphamate‡ | 20 | 15 | 15 | 6 | 1 | 0 | 1/15 | 0·7 |
| Ammonium 2-naphthylamine-N-glucosiduronate | 20 | 13 | 9 | 5 | 1 | 0 | 1/13 | 0·5 |
| 2-Amino-6-naphthol hydrochloride‡ | 20 | 13 | 11 | 4 | 1 | 0 | 1/13 | 0·5 |
| 2-Acetamido-6-naphthol‡ | 19 | 11 | 10 | 0 | 0 | 0 | 0/11 | — |
| 2-Amino-1-naphthol hydrochloride | 25 | 13 | 13 | 0 | 1 | 0 | 1/13 | 0·5 |
| 2-Amino-1-naphthyl glucosiduronic acid | 25 | 17 | 17 | 0 | 4 | 3 | 7/17 | 0·01 |
| 1-Dimethylamino-2-naphthol | 20 | 14 | 11 | 0 | 1 | 1 | 2/14 | 0·3 |
| 1 : 2-5 : 6-Dibenzphenazine | 20 | 11 | 11 | 10 | 1 | 0 | 1/11 | 0·5 |
| *Amines and aminophenols* | | | | | | | | |
| 4-Dimethylamino-3-hydroxydiphenyl | 20 | 13 | 11 | 0 | 1 | 1 | 2/13 | 0·3 |
| 4-Dimethylaminoazobenzene | 20 | 16 | 14 | 0 | 1 | 0 | 1/16 | 0·7 |
| 2 : 6-Diamino-3-phenylazopyridine | 20 | 14 | 12 | 10 | 2 | 2 | 4/14 | 0·03 |
| 2-Amino-4 : 5-dimethylphenol | 25 | 18 | 18 | 0 | 0 | 5 | 5/18 | 0·02 |
| 8-Hydroxyquinoline | 20 | 16 | 13 | 0 | 2 | 3 | 5/16 | 0·01 |
| *Tryptophan derivaties* | | | | | | | | |
| 3-Hydroxykynurenine | 35 | 25 | 10 | 4 | 1 | 6 | 7/25 | 0·02 |
| Kynurenic acid | 20 | 12 | 11 | 0 | 0 | 1 | 1/12 | 0·3 |
| 8 - Methoxy - 4 - hydroxyquinoline - 2 - carboxylic acid | 30 | 19 | 14 | 0 | 1 | 3 | 4/17 | 0·08 |
| 3-Hydroxyanthranilic acid | 60 | 40 | 39 | 24 | 3 | 8 | 11/40 | 0·02 |
| 3-Hydroxyanthranilic acid‡ | 30 | 12 | 19 | 5 | 0 | 1 | 1/22 | 1·0 |
| Methyl-3-hydroxyanthranilate | 20 | 13 | 13 | 9 | 0 | 2 | 2/13 | 0·3 |
| 2-Amino-3-hydroxy acetophenone | 25 | 17 | 17 | 0 | 1 | 4 | 5/17 | 0·03 |
| 5-Hydroxytryptamine creatinine sulphate | 20 | 14 | 14 | 10 | 1 | 0 | 1/14 | 0·8 |

‡ Pellets made with paraffin wax in place of cholesterol.

bearing mice to the number of mice surviving for 30 weeks. The probability that this ratio differed from the incidence in the control series was calculated by the $\chi^2$ test.

Although no tumours were seen in the 13 mice which survived for 30 weeks or more after treatment with paraffin wax pellets, one mouse killed after 52 weeks showed hyperplastic changes of the bladder epithelium. Of the 24 mice surviving 30 weeks or more after implantation with cholesterol pellets one had a carcinoma and another mouse revealed hyperplastic changes. Thus the control treatment does not produce a completely negative result. Of the 26 groups of mice treated with substances under test, 9 contained one tumour in each group and except for the 3-hydroxy-4-aminodiphenyl group in which only 10 mice survived 30 weeks the substances implanted in these groups may be considered as inactive. These include :

1 : 2–5 : 6-Dibenzanthracene-3 : 4-quinone
2-Phenylphenanthrene-3 : 2′-dicarboxylic acid
Potassium 2-naphthylsulphamate
Ammonium-2-naphthylamine-N-glucosiduronate
2-Amino-6-naphthol hydrochloride
2-Acetamido-6-naphthol
1-Dimethylamino-2-naphthol,
4-Dimethylaminoazobenzene.

Examples of the lesions produced are illustrated in Fig. 3–8.

### DISCUSSION

The technique of implanting pellets into the bladders of mice can be used to indicate whether the carcinogenic activity is due to the substance itself or to a metabolite, because the possibilities of metabolic changes occurring in the bladder are limited. The present results are considered under five headings, three of which are concerned with the problems of the role of metabolism of carcinogens in relation to their biological action.

### 1. *Miscellaneous substances*

Maleic hydrazide was tested in the present experiments because Darlington and McLeish (1951) had found it to be a mitotic poison for plant cells. It did not induce cancer in the bladder and this is in agreement with the findings of Barnes and Haddow (personal communication) who did not obtain a significant incidence of tumours in rats injected with this compound. Although it produced chromosome damage in plant cells, it does not interfere with mitosis in cells of the Walker carcinoma, when injected into tumour-bearing rats (Boyland and Koller, unpublished observations).

Pellets containing xanthine induced carcinomata. This is in agreement with earlier work (Haddow, personal communication) in which injection of xanthine induced sarcomata in rats.

Pellets containing saccharin induced a significant incidence of bladder tumours. On the other hand, in an experiment in which 20 mice were injected twice weekly with 1 g. saccharin per kg. body weight for 12 weeks, no tumours developed, although 12 mice lived for one year and 8 mice were killed 2 years after the

Maleic hydrazide

Xanthine

Saccharin

Formulae A

commencement of the experiment. The bladders of these mice were examined *post-mortem* as in the bladder implantation experiments. In further experiments carried out with Professor P. C. Koller, the injection of 1 g. per kg. of saccharin as the sodium salt into rats bearing the Walker Carcinoma produced no apparent chromosome damage. Fitzhugh, Nelson and Frawley (1951) noticed that feeding of saccharin to rats was associated with " an increased incidence of the ordinarily uncommon condition of abdominal lymphosarcoma " but these authors did not consider that saccharin was carcinogenic. Salaman and Roe (1956) found that saccharin had some initiating action, that is, it caused skin tumours to appear in mice when its application was followed by treatment with croton oil. The induction of bladder tumours with saccharin suggests that the presence of the solid pellet in the bladder may have a promoting action and that the method of bladder implantation detects incomplete carcinogens.

2. 1 : 2–5 : 6-*Dibenzanthracene and derivatives*

Bladder tumours were produced with 1 : 2–5 :6-dibenzanthracene but the oxidation product 1 : 2–5 : 6-dibenzanthracene-3 : 4-quinone and the metabolite 2-phenylphenanthrene-3 : 2′-dicarboxylic acid gave negative results. The parent compound thus appears to initiate a neoplastic change without biochemical elaboration. If this is the case, then the conversion of 1 :2–5 : 6-dibenzanthracene into 2-phenylphenanthrene-3 : 2′-dicarboxylic acid described by Bhargava *et al.* (1955) if of the nature of a side reaction or detoxication process. The possibility remains that the phenylphenanthrene dicarboxylic acid cannot penetrate into cells, but can be produced by metabolism of 1 : 2–5 : 6-dibenzanthracene

1 : 2-5 : 6 Dibenzanthracene

1 : 2-5 : 6 Dibenzanthracene
3 : 4-quinone

2-Phenylphenanthrene
3 : 2′-dicarboxylic acid

Formulae B—Hydrocarbon derivatives after the latter has entered susceptible cells. The simpler explanation is, however, that the carcinogenic hydrocarbons are carcinogenic *per se*. The carcinogenic action of hydrocarbons could be due to their ability to form complexes with the purines of nucleic acid and " this association may change the nucleic acid sufficiently for chromosome aberrations to result " (Boyland, 1952). The induction of tumours with the hydrocarbon is in agreement with the findings that the polycyclic compounds 20-methylcholanthrene and 3 : 4–5 : 6-dibenzcarbazole are carcinogenic in the mouse bladder (Bonser, Clayson, Jull and Pyrah, 1952).

3. *Derivatives of 2-naphthylamine*

Unlike 1 : 2–5 : 6-dibenzanthracene, some aromatic amines appear to be indirect carcinogens which must be metabolised into active forms. 2-Naphthylamine itself possesses only very slight activity as a bladder carcinogen but 2-amino-1-naphthol is active (Bonser, Bradshaw, Clayson and Jull, 1956) and of the seven derivatives examined only 2-amino-1-naphthyl glucosiduronic acid produced a significant incidenced of tumours. It had been argued (Boyland and Manson, 1955; Boyland, 1956) that of the 16 identified metabolites of 2-naphthylamine listed in Table I only one—2-amino-1-naphthyl glucosiduronic acid—should be carcinogenic and this compound has now been shown to induce tumours. The relatively high proportion of papillomas (4 papillomas, 3 carcinomas) induced with this metabolite is remarkable. If one assumes that the papillomas would have developed into carcinomas in time then this finding suggests that the substance is slow in action.

The insignificant incidence of tumours induced with 2-amino-6-naphthol hydrochloride an 2-acetamido-6-naphthol is of interest as these are representatives of the type of oxidation product in which the oxygen has entered the benzene ring remote from the amino group. The inactivity of these substances makes it improbable that derivatives of these (e.g., Nos. 9, 10, 11 and 12 of Table I) would be carcinogenic but nevertheless they are being tested in experiments which are now in progress. The inactivity of 2-amino-6-naphthol compared with the activity of 2-amino-1-naphthol which is an *ortho* aminophenol is in agreement with the idea that the carcinogenic activity of some at least of the aromatic amines is dependent on their metabolic conversion to *ortho* aminophenols. The susceptibility of the dog and man to the carcinogenic effect of 2-naphthylamine is probably related to the relatively high proportion of 2-amino-1-naphthol formed *in vivo* by these species. In rodents, which are more resistant to 2-naphthylamine, derivatives of 2-amino-6-naphthol appear to be the predominant metabolites (Clayson, 1953). 2-Amino-1-naphthol, however, does not usually occur free in the urine but conjugated with glucuronic acid and sulphuric acid. In the presence of $\beta$-glucuronidase the glucosiduronate may be hydrolysed, liberating the active compound. In the present experiment, 2-amino-1-naphthol glucosiduronic acid produced more tumours than did the free 2-amino-1-naphthol; possibly because the carcinogenic activity of the latter was in part masked by toxic effects. When the pellet of 2-amino-1-naphthol in cholesterol was inserted into the bladder, a dose toxic to proliferating cells might have been introduced, whereas in the case of the glucuronic acid conjugate, the free carcinogen would be liberated slowly by $\beta$-glucuronidase in the urine.

Bonser *et al.* (1956) has shown that 1-amino-2-naphthol and also 2-amino-1-naphthol were carcinogenic in the bladder of mice. Our results suggest that the substitution of the amino group as in 1-dimethylamino-2-naphthol destroyed the activity. This is surprising as usually methylation of carcinogenic aromatic amines either in the amino group itself or in the positions *ortho* to the amino group enhances their carcinogenic activity.

4. *Amines and aminophenols*

4-Dimethylamino-3-hydroxydiphenyl, which is the *ortho* aminophenol corresponding to 4-dimethylaminodiphenyl which had been shown to be carcinogenic in rats (Miller, Miller, Sandin and Brown, 1949), gave an indefinite result (2 tumours in 13 mice).

2-Amino-4 : 5 dimethylphenole

8-Hydroxyquinoline

4 Dimethylamino-3-hydroxy diphenyl

4-Dimethylaminoazobenzene

Pyridium
(2 : 6-Diamino-3-phenylazopyridine)

3-(4′-Dimethylaminophenyl)azopyridine
N-oxide

Formulae C—Amines and Aminophenols

4-Dimethylaminoazobenzene or Butter Yellow is a liver carcinogen which was inactive in the bladder. 2 : 6-Diamino-3-phenylazopyridine hydrochloride (Pyridium) which is used as a urinary analgesic induced bladder tumours. This compound is similar to 3-(4′dimethylaminophenyl) azopyridine-N-oxide and other phenylazopyridine derivatives which Brown, Malloy, McCarthy, Verrett and Cerecedo (1954) found to be hepatic carcinogens, and is thus a heterocyclic analogue of dimethylaminobenzene. In this compound, chelation is possible through the 2-amino group and the azo linkage but dimethylaminoazobenzene which is not a bladder carcinogen could be metabolised to a hydroxy-derivative with chelating properties.

2-Amino-4 : 5-dimethylphenol (2-amino-4 : 5-xylenol) is the simplest aminophenol found to be carcinogenic. Aminophenol itself and monomethyl derivatives are now being tested by the bladder implantation technique although Miller and Miller (1948) found *ortho* aminophenol to be non-carcinogenic when tested by feeding to rats.

A number of *ortho* aminophenols are now known to induce cancer in the bladder of mice and the problem of the mechanism of action presents itself. These aminophenols are very reactive compounds being readily oxidised and combining with many reagents. They are also chelating agents forming complexes with metals (Charles and Freiser, 1952). For this reason the active chelating agent, 8-hydroxyquinoline or oxine, was tested and gave tumours in 6 out of 16 mice which survived 30 weeks. Thus it is possible that this compound and the *ortho* aminophenols are active by virtue of their chelating power. The activity might therefore be due to combination of the carcinogen with the desoxyribonucleic acid (DNA) of chromosomes through the metals of the DNA forming a double chelate as a distorted DNA molecule. Other possibilities are that the chelating agent competes with the DNA molecules for metals which are probably essential for the correct functioning of DNA of chromosomes or that they break nucleoprotein molecules as observed by Kirby (1956) *in vitro*. On the other hand the fact that Bonser *et al.* (1954) found 1-methoxy-2-naphthylamine induced bladder cancer in mice is not in agreement with this hypothesis as this compound should be devoid of chelating activity.

The carcinogenic action of 8-hydroxyquinoline is of practical interest because this substance is used as a spermicidal agent in contraceptive preparations and as a preservative particularly in tobacco in Germany (Wegner, 1955). Hoch-Ligeti (1956) has described the induction of carcinoma of the vagina in rats treated intravaginally with a contraceptive cream containing 8-hydroxyquinoline. That there might be some relation between spermicidal and carcinogenic action is perhaps not surprising as both effects are probably concerned with nuclear poisoning. In view of this and the result obtained with 8-hydroxyquinoline, a number of spermicidal agents are being tested for carcinogenic activity in the vagina of the mouse.

5 *Tryptophan metabolites*

The natural history of bladder cancer in men working with aromatic amines does not differ from that of bladder cancer occurring iu the general population. In the case of the occupational cancer the cause would seem to be the *ortho* aminophenols liberated in the urine by the action of urinary $\beta$-glucuronidase on excreted metabolites of aromatic amines such as 2-naphthylamine and 4-aminodiphenyl. One might expect therefore that bladder cancer in the general population is due to an excreted carcinogen. In normal metabolism in man and animals tryptophan is converted to nicotinic acid by way of kynurenine, 3-hydroxykynurenic and 3-hydroxy anthranilic acid ; 3-hydroxykynurenine and 3-hydroxyanthranilic acid are *ortho* aminophenols and they are often present in human urine. 2-Amino-3-hydroxyacetophenone is another *ortho* aminophenol derived from tryptophan which is sometimes present in human urine (Dalgleish, 1955). All these naturally-occurring *ortho* aminophenols induced bladder tumours in our experiments.

Although pellets of 3-hydroxyanthranilic acid in cholesterol produced tumours, pellets of the same compound in paraffin wax did not induce a significant number of tumours (1 tumour in 22 mice). The diffusion of 3-hydroxyanthranilic acid from pellets was therefore examined. The pellets were incubated in 1 ml. water at 38°. After different intervals (indicated by the points in the figure) the aqueous phase was replaced and the 3-hydroxyanthranilic acid estimated by measurement of

TABLE I—*cont.*

Formulae D—Tryptophan Metabolites the absorption at 298 m$\mu$ in 0·1 N-HCl solution using a Unicam Spectrophotometer. The results (Fig. 2) show that the rate of diffusion of 3-hydroxyanthranilic acid is such that about half of the 2-mg. of 3-hydroxyanthranilic acid originally present diffused out of a cholesterol pellet in 15 days and that about 10 per cent diffused out in the first 24 hours. The rate of diffusion falls with time and varies so that the diffusion at any time is proportional to the square of the amount of 3-hydroxyanthranilic acid present at that time. On the other hand only 6 per cent of the 3-hydroxyanthranilic acid was detected in the water in which paraffin wax pellets containing the acid had been incubated for 10 days.

FIG. 2.—Release of 3-hydroxyanthranilic acid (3HAA) from (A) one cholesterol pellet containing 2 mg. 3HAA and (B) ten paraffin pellets each containing 2 mg. 3HAA.

Diffusion of the carcinogens with urine in the bladders of the mice would presumably occur at comparable rates and this could explain the apparent inactivity of the 3-hydroxyanthranilic acid in paraffin wax pellets. The diffusion of substances from paraffin wax must depend on the material first dissolving in the continuous phase of wax and then dissolving out with the surrounding water. Diffusion out must therefore depend on solubility in wax and in water.

Diffusion from cholesterol pellets can proceed either by solution in the sterol or by solution in water which penetrates between the crystals in the pellet. Polar substances which are soluble in water are therefore likely to diffuse from the compressed cholesterol pellets, but not from pellets of paraffin wax. On the other hand substances which are readily soluble in water may diffuse out of cholesterol pellets too quickly, so that they either produce toxic effects or are rapidly excreted.

The inactivity of 2-amino-1-naphthol hydrochloride in the present experiments, in contrast to the activity of this substance in paraffin wax found by Bonser *et al.* (1956) might be due to the compound diffusing too rapidly from the cholesterol pellets.

In view of these considerations the behaviour of different substances in pellets should be studied and the appropriate medium—either paraffin wax, cholesterol or other material—chosen. The medium should allow the suspected carcinogen to be released at a suitable rate.

In these experiments there is the possibility that the action is due to some active impurity. Although attempts were made to use pure materials, many of the substances used are unstable. Thus, 3-hydroxyanthranilic acid is easily oxidised to 2-aminophenoxazone-4 : 5-dicarboxylic acid acid (Butenandt, Biekert and Neubert, 1957) and this might be a contaminant of the acid, Similarly 3-hydroxy-2-aminoacetophenone is readily oxidised to 3-amino-4 : 5-diacetyl-phenoxazone (2). These phenoxazones are similar in structure to the chromophore of actinomycin and the amnochrome pigments of insects. Derivatives of this type are under investigation for carcinogenic activity.

Plaine and Glass (1955) found that the addition of *l*-tryptophan indole or anthranilic acid to the diet of *Drosophila melanogaster* larvae produced an enormous increase in the incidence of tumours in the larvae. The effect which was even greater when the larvae were also exposed to oxygen at the same time, may be connected with the carcinogenic action of tryptophan metabolites in the bladders of mice.

Estimations of the excretion of 3-hydroxykynurenine and 3-hydroxyanthranilic acid by human subjects has shown that men with cancer of the bladder excrete more of these substances than do patients with other diseases (Boyland and Williams, 1956). These aminophenols are probably excreted in urine in conjugated forms including the glucuronides and sulphuric esters which should, however, be hydrolysed by $\beta$-glucuronidase or sulphatase in the urine, and the urine of patients with cancer of the bladder usually contains abnormally high $\beta$-glucuronidase and sulphatase activity (Boyland, Wallace and Williams, 1955).

The induction of bladder cancer in man by this mechanism is thus thought to be dependent on the release of carcinogenic *ortho* aminophenols in urine from inactive conjugated precursors by the action of enzymes. The effect can therefore be expected to depend on (1) the concentration of the *ortho* aminophenol glucosiduronide, (2) the activity of $\beta$-glucuronidase and sulphatase in the urine and (3) the time during which the urine remains in the bladder with enzymes acting on the excreted aminophenol conjugates and liberating the carcinogenic *ortho* aminophenols.

All these factors can be reduced unspecifically by increasing the water consumption and so diluting the urine. The first factor can be reduced by avoiding contact with aromatic amines or other precursors of *ortho* aminophenols or in some cases by correcting the diet so that phenolic metabolites of tryptophan are not excreted. The second or enzymic factor can be reduced by treatment with 1 : 4-saccharolactone which is a potent inhibitor of $\beta$-glucuronidase (Levvy, 1952).

### SUMMARY

(1) The operation for implantation of pellets into the bladders of mice has been modified and used to test substances for their carcinogenic activity.

(2) Xanthine and saccharin induced bladder tumours, but maleic hydrazide did not under these conditions.

(3) Under conditions in which 1 : 2–5 : 6-dibenzanthracene gave bladder tumours, 1 : 2–5 : 6-dibenzanthracene-3 : 4-quinone and a metabolite of the hydrocarbon, 2-phenylphenanthrene-2′ : 3-dicarboxylic acid did not induce tumours, indicating that this metabolite is not concerned in carcinogenesis.

(4) Of the sixteen metabolites which have been identified in urine of animals dosed with 2-naphthylamine, five have been tested in the mouse bladder and only one of these, 2-amino-1-naphthol glucosiduronic acid gave tumours. This is in agreement with knowledge of the behaviour of these substances.

(5) Of the simple aminophenols tested, 2-dimethylaminophenol and 2-amino-4 : 5-dimethylphenol produced tumours. 4-Dimethylaminoazobenzene (DAB) did not induce tumours, but Pyridium (2 : 6-diamino-3-phenylazopyridine) was active.

(6) 8-Hydroxyquinoline, which like the *ortho* aminophenols is a chelating agent (which is used as spermicide and fungicide) induced cancer in the mouse bladder.

(7) Three *ortho* aminophenols which are metabolites of tryptophan—2 amino 3-hydroxyacetophenone, 3-hydroxykynurenine and 3-hydroxy anthranilic acid induced cancer on implantation in the bladders of mice. The possibility of such compounds being the cause of cancer of the bladder in man is discussed.

We are indebted to Dr. A. L. Morrison of Roche Products for the gift of xanthurenic acid and to Dr. D. Manson and Dr. P. Sims for a number of compounds used. We should like to thank Miss M. Biggs, Mr. J. W. Gorrod, Mr. R. E. S. Prout and Mr. E. Woollard for skilled technical assistance and to Mr. K. G. Moreman, A.R.P.S., A.I.B.P., for the photographs. The work has been supported by grants to the Chester Beatty Research Institute (Institute of Cancer Research : Royal Cancer Hospital) from the British Empire Cancer Campaign, the Jane Coffin Childs Memorial Fund for Medical Research, the Anna Fuller Fund, and the National Cancer Institute of the National Institutes of Health, U.S. Public Health Service.

### REFERENCES

Bhargava, P. M., Hadler, H. I. and Heidelberger, C.—(1955) *J. Amer. chem. Soc.*, **77**, 2877.

Bonser, G. M., Bradshaw, L., Clayson, D. B. and Jull, J. W.—(1956) *Brit. J. Cancer*, **10**, 531.

*Idem*, Clayson, D. and B. Jull, J. W.—(1951) *Lancet*, ii, 286.—(1954) *Ann. Rep. Brit. Emp. Cancer Compgn*, **31**, 192.

*Idem*, Clayson, D. B., Jull, J. W. and Pyrah, L. N —(1952) *Brit. J. Cancer*, **6**, 412. —(1953) *Ibid.*, **7**, 456.

*Idem* and Jull, J. W.—(1956) *J. Path. Bact.*, **72**, 489.

Boyland, E.—(1952) *Cancer Res.*, **12**, 77.—(1956) *Bull. Soc. Chim. biol., Paris*, **38**, 827.

*Idem* and Manson, D.—(1955) *Biochem. J.*, **60**, ii.—(1957) *Ibid.* (in press).

*Idem*, Manson, D. and Orr, S. F. D.—(1957) *Ibid.*, **65**, 117.

*Idem*, Manson, D., Sims, P. and Williams, D. C.—(1956) *Ibid.*, **62**, 68.

*Idem* and Sims, P.—(1954) *J. chem. Soc.*, 980.

*Idem*, Wallace, D. M. and Williams, D. C.—(1955) *Brit. J. Cancer*, **9**, 62.

*Idem* and Watson, G.—(1956) *Nature*, **177**, 837.

*Idem* and Williams, D. C.—(1956) *Biochem. J.*, **64**, 578.

Brown, E. V., Malloy, P. C., McCarthy, P., Verret, M. J. and Cerecedo, L. R.—(1954) *Cancer Res.*, **14**, 715.
Butenandt, A. and Hellmann, G.—(1950) *Z. Naturf.*, B**56**, 445.
*Idem*, Biekert, E. and Neubert, G.—(1957) *Ann. Chem.*, **602**, 72.
Charles, R. G. and Freiser, W.—(1952) *J. Amer. chem. Soc.*, **74**, 1385.
Clayson, D. B.—(1953) *Brit. J. Cancer*, **7**, 460.
Dalgleish, C. E.—(1955) *Biochem. J.*, **61**, 334.
Darlington, C. D. and McLeish, J.—(1951) *Nature*, **167**, 407.
Fitzhugh, O. G., Nelson, A. A. and Frawley, J. P.—(1951) *J. Amer. pharm. Assoc.*, Sci. Ed., **40**, 583.
Hoch-Ligeti, C.—(1956) *Proc. Amer. Ass. Cancer Res.*, **2**, 118.
Jull, J. W.—(1951) *Brit. J. Cancer*, **5**, 328.
Kirby, K. S.—(1956) *Biochem. J.*, **62**, 31P.
Levvy, G. A.—(1952) *Ibid.*, **52**, 464.
Maisin, J. and Picard, E.—(1924) *C.R. Soc. Biol., Paris*, **91**, 799.
Miller, J. A. and Miller, E. C.—(1948) *J. exp. Med.*, **87**, 139.
*Idem*, Miller, E. C., Sandin, R. B. and Brown, R. K.—(1949) *Cancer Res.*, **9**, 504.
Oppenheimer, B. S., Oppenheimer, E. T., Danishefsky, I., Stout, A. P. and Eirch, F. R.—(1955) *Ibid.*, **15**, 333.
Picard, E.—(1927) *Ann. Soc. sci. Brux.*, Ser. C., **47**, 148.
Plaine, H. L. and Glass, B.—(1955) *J. Genet.* **53**, 244.
Rudali, G., Chalvet, H. and Winternitz, F.—(1955) *C.R. Acad. Sci., Paris*, **240**, 1738.
Salaman, M. H. and Roe, F. J. C.—(1956) *Brit. J. Cancer*, **10**, 363.
Wegner, E.—(1955) *Z. LebensmittUntersuch.*, **102**, 34.

### EXPLANATION OF PLATES

Fig. 3.—Papilloma from bladder of a mouse 40 weeks after implantation of cholesterol pellet containing 2-amino-1-naphthol glucosiduronic acid. × 25.

Fig. 4.—Section of distended bladder from a mouse 40 weeks after implantation of cholesterol pellet containing 2-amino-3-hydroxyacetophenone. × 10.

Fig. 5.—Higher magnification of focus of carcinoma from bladder shown in Fig. 4, showing small invasive focus of carcinoma. × 40.

Fig. 6.—Section of bladder of a mouse killed 40 weeks after implantation of a cholesterol pellet containing 3-hydroxyanthranilic acid showing solid differentiated carcinoma. × 11.

Fig. 7.—Section of bladder shown in Fig. 6. × 65.

Fig. 8.—Macroscopic view of the urinary bladder of a mouse killed 52 weeks following implantation of 3-hydroxyanthranilic acid in cholesterol showing papillary carcinoma. Four large carcinomatous lesions are seen projecting into the lumen of the bladder. × 3½.

3

4

5

Allen, Boyland, Dukes. Horning and Watson.

6

8

7

Allen, Boyland. Dukes, Horning and Watson.